# *Ankfn1*-mutant vestibular defects require loss of both ancestral and derived paralogs for penetrance in zebrafish

**DOI:** 10.1093/g3journal/jkab446

**Published:** 2021-12-25

**Authors:** Kevin D Ross, Jie Ren, Ruilin Zhang, Neil C Chi, Bruce A Hamilton

**Affiliations:** 1 Biomedical Sciences Graduate Program, University of California San Diego, La Jolla, CA 92093, USA; 2 Department of Medicine, University of California San Diego, La Jolla, CA 92093, USA; 3 Rebecca and John Moores UCSD Cancer Center, University of California San Diego, La Jolla, CA 92093, USA; 4 Institute for Engineering in Medicine, University of California San Diego, La Jolla, CA 92093, USA; 5 Institute for Genomic Medicine, University of California San Diego, La Jolla, CA 92093, USA; 6 Department of Cellular and Molecular Medicine, University of California San Diego, La Jolla, CA 92093, USA

**Keywords:** *Ankfn1*, *Ankfn1-like*, *wide awake*, *Banderuola*, *nmf9*, paralogy, penetrance, domain architecture

## Abstract

How and to what degree gene duplication events create regulatory innovation, redundancy, or neofunctionalization remain important questions in animal evolution and comparative genetics. *Ankfn1* genes are single copy in most invertebrates, partially duplicated in jawed vertebrates, and only the derived copy retained in most mammals. Null mutations in the single mouse homolog have vestibular and neurological abnormalities. Null mutation of the single *Drosophila* homolog is typically lethal with severe sensorimotor deficits in rare survivors. The functions and potential redundancy of paralogs in species with two copies are not known. Here, we define a vestibular role for *Ankfn1* homologs in zebrafish based on the simultaneous disruption of each locus. Zebrafish with both paralogs disrupted showed vestibular defects and early lethality from swim bladder inflation failure. One intact copy at either locus was sufficient to prevent major phenotypes. Our results show that vertebrate *Ankfn1* genes are required for vestibular-related functions, with at least partial redundancy between ancestral and derived paralogs.

## Introduction

*Ankfn1* is a recently annotated gene with an interesting genealogy (Zhang et al. 2015). Orthologous genes are recognized by the eponymous ankyrin (ANK) and fibronectin type 3 (FN3) motifs and three highly conserved nonmotif domains. Orthologs are found in all animal lineages studied to date except urochordates and in some sister groups to animals, including choanoflagellates and filastereans. Some unicellular examples encode an aminoterminal CRIB domain. Some unicellular and nearly all invertebrate homologs include a carboxyterminal Ras association (RA) domain. The orthology group shows a single member per genome outside of vertebrates. In an ancestor to jawed vertebrates, the ancestral gene was incompletely duplicated, with the derived paralog losing the RA domain. In an ancestor to therian mammals, the ancestral copy was lost. All animal genomes analyzed to date thus have zero (urochordates), one ancestral (all other invertebrates), one derived (therian mammals), or two (other vertebrates) *Ankfn1* genes.

*Ankfn1* mutations were identified independently by three groups using forward genetics in flies and mice. Transposon insertion mutations in *Drosophila* (*wake* alleles) were recovered in a screen for sleep-related phenotypes (Liu et al. 2014). Although originally reported as null, these appear to have been isoform-specific alleles, which prevented expression in some tissues. An RNAi screen to identify genes required for asymmetric cell division in *Drosophila* sensory organ precursor cells identified the same gene as an essential regulator of Numb segregation during cell division; null mutations (*Banderuola* alleles) had severe developmental consequences and poor viability (Mauri et al. 2014). Banderuola protein interacted physically with Discs large, and inhibition of both genes elicited tumorigenesis in the *Drosophila* brain. We identified a presumptive null allele of the mouse homolog by positional cloning of a neurological mutation (*nmf9*) marked by vestibular and neurological phenotypes (Zhang et al. 2015). Subsequent alleles made by genome editing confirmed gene identification by noncomplementation and showed the functional importance of a nonmotif domain including a highly conserved GLYLGYLK peptide sequence, with even a glycine-to-alanine substitution failing to complement the original mutation. *Nmf9* mice were viable and fertile but had several neurological abnormalities—including defects in circadian onset, fear learning, and vestibular function. We also showed that, in *Drosophila* frame-shift mutations in distinct domains, all had *Banderuola*-like phenotypes. The few flies that survived to adulthood had profound sensorimotor deficits and died prematurely. Flies heterozygous for null mutations had abnormal sleep patterns, broadly consistent with *wake* mutants and demonstrating dosage sensitivity for some phenotypes.

Differences between flies and mice both in domain architecture and in phenotypic severity raise questions about essentiality and conservation of *Ankfn1* function in other animals, especially vertebrate groups that have paralogous copies. This difference between mammals and other vertebrates is relatively common: ∼15% of human genes are single copy in humans and have more than one identified homolog in the zebrafish *Danio rerio* (Howe et al. 2013). Paralogous genes can have variable functional outcomes. Full redundancy can result in one copy decaying into a pseudogene that has lost function relative to the ancestral gene (Mighell et al. 2000). Duplication can also lead to new or divergent functions, through neofunctionalization or subfunctionalization (Force et al. 1999). The facile genetics of *D. rerio* makes it an ideal system to test *Ankfn1* paralogous gene function in the typical vertebrate arrangement of one ancestral and one derived copy. The ancestral copy with an intact RA domain is on chromosome 24, while the derived copy without RA is on chromosome 12. Assessing mutations of each homolog independently and in combination should allow direct tests of essentiality, functional redundancy, and possible neofunctionalization after duplication (Ohno 1970).

Here, we provide new resolution on the evolution and constraint of the *Ankfn1* gene genealogy and use CRISPR/Cas9 to generate mutations in both *D. rerio* homologs to define major functions and test the degree of genetic redundancy between paralogs in a nonmammal vertebrate. We generated a frameshifting deletion allele of the derived chromosome 12 paralog (*Ankfn1*), and both a frameshifting insertion allele and an in-frame deletion allele at the GLYLGYLK site of the ancestral chromosome 24 paralog (*Ankfn1-like*). We observed mutant progeny among digenic crosses and identified vestibular-related phenotypes that were highly penetrant in fish with biallelic inactivation at both loci, weakly penetrant in fish with only the derived copy inactivated, and indistinguishable from background in fish with only the ancestral copy inactivated. Affected fish had severe locomotor and balance deficits as larvae, did not inflate their swim bladder by 5 days postfertilization, and died prior to adulthood. Swim bladder inflation requires vestibular function to orient larvae in the water column to gulp air at the air–water interface and perturbation of utricular otoliths prevents normal inflation (Riley and Moorman 2000; Lindsey et al. 2010), providing a consistent interpretation for observed phenotypes centered on previously reported inner ear expression of both paralogs (Zhang et al. 2015). These results suggest genetic overlap between the ancestral and derived homologs for essential functions, including development of the vestibular system.

## Materials and methods

### Sequence analysis

Previously reported *Ankfn1* homologs (Zhang et al. 2015) were updated to more recent annotations and supplemented with new homologs identified through iterative BLASTP searches (Altschul et al. 1990) targeting previously unavailable species. Only one species per genus was included unless species showed 10% or greater nonidentity between orthologs (*Drosophila* species group). Gene models annotated as low quality were excluded. Domain annotation for each included sequence was characterized using SMART (Letunic and Bork 2018; Letunic et al. 2021). For nonmammal vertebrates, inclusion required each paralog to include their expected domain composition. Species, accession numbers, taxonomic groups, and annotated domains for each included sequence are provided in Supplementary Table 1 and FASTA-formatted sequences are provided in Supplementary Data 1. Curated homologs were aligned using MUSCLE (Edgar 2004a, 2004b) from the European Bioinformatics Institute web portal (https://www.ebi.ac.uk/Tools). Alignment files for Holozoa, nontherian vertebrates, and nontherian vertebrate ancestral and derived subsets are provided in Supplementary Data 2–5, respectively. Sequence conservation of aligned residues was evaluated using Valdar’s scoring method in ScoreCons (Valdar 2002) and using Jensen–Shannon divergence (Capra and Singh 2007). Physicochemical and evolutionary constraints on specific positions were assessed using the median replacement score in the multivariate analysis of protein polymorphism (MAPP) (Stone and Sidow 2005). Predicted ANKFN1 structure was accessed and visualized from the European Bioinformatics Institute web interface https://alphafold.ebi.ac.uk/.

### Genome editing

Mutant fish were generated by injecting a cocktail of sgRNAs and Cas9 mRNA into *D.rerio* strain AB one-cell-stage embryos (Jao et al. 2013). *Ankfn1*-homolog sgRNAs were in vitro transcribed from PCR-amplified templates (Supplementary Table 2). Synthetic Cas9 mRNA was transcribed from pCS2-nCas9n (gift of Dr. Wenbiao Chen, Addgene plasmid # 47929). The injection cocktail contained 200 ng/μl Chr12 sgRNA, 300 ng/μl Chr24 sgRNA, and 300 ng/μl Cas9 mRNA. Founder mutations were identified by pooling G1 embryos from individual G0-injected fish and screening for length polymorphisms by PCR. PCR products from putative mutants were Sanger sequenced to identify specific alleles. Husbandry and stock maintenance were carried out at 28°C as described (Zhang et al. 2013; Han et al. 2016).

### Breeding and epistasis

G0 founders were outcrossed to wild-type fish to produce G1 offspring. Animals in cross 1 were G2 offspring from an intercross of G1 mutants. Crosses 2 and 3 were G4 and G5 fish from intercross matings. Balance and motor coordination were assessed by observing fish larvae following the initiation of a startle response by tapping the side of the Petri dish or gentle stimulation of the tail with a pipette as described (Kwak et al. 2006). Larvae were categorized as unaffected or affected based on subsequent swim bladder inflation or failure to inflate by day 5 postfertilization. After phenotyping, single zebrafish larvae were harvested into individual wells in DNA extraction buffer for use in genotyping assays.

### PCR genotyping

DNA was extracted from single larvae in 50 µl of 50 mM NaOH for 20 min at 95°C and then neutralized with 5 µl of 1 M Tris-HCl, pH 8.3. PCR was performed in 20 µl volume. Each reaction contained 1× PCR buffer (10 mM Tris, pH 9.0, 2 mM MgCl_2_, 50 mM KCl, 0.1% Triton X-100), 0.27 µM each primer, 140 mM each dNTP, 1.2 U Taq DNA polymerase, and 1 µl of zebrafish DNA suspension was prepared on ice. PCR thermal cycler program was 95°C for 1 min followed by 35 cycles of (95°C 20 s, 55°C 20 s, 72°C 30 s), 72°C for 3 min, and 12°C for up to 30 min. Electrophoresis was performed on 4% agarose gels with ethidium bromide in 1× TBE and visualized by 365 nm UV transillumination. Primers for genotyping are given in Supplementary Table 3.

## Results

### A highly constrained peptide is a predicted structural element of ANKFN1 proteins

Previous analysis of 14 diverse metazoan *Ankfn1* homologs identified strong conservation of ANK, FN3, and three nonmotif domains based on d*N*/d*S* analysis across sliding windows (Zhang et al. 2015). To better understand conserved features in ANKFN1 proteins, we curated additional homologs, many of which were unavailable or poorly annotated at the time of the earlier analysis (Supplementary Table 1). Surprisingly, annotation of two monotreme species (*Ornithorhynchus anatinus* and *Tachyglossus aculeatus*) included ancestral paralogs, although the *Tachyglossus* ancestral paralog appeared incomplete and was not used beyond confirmation of paralogous genes in egg-laying mammals. Placental and marsupial species each showed only a single copy of the derived paralog. These observations support a date for loss of the ancestral paralog in the lineage leading to therian mammals, rather than to all mammals. Gene models from several ray-finned fish (Actinopterygii) lineages support a third, lineage-restricted paralog with a more diverged protein sequence that has weaker match to the ANK motifs, GLYVGILK in place of the GLYLGYLK peptide, and no RA domain. However, neither *Danio* nor closely related *Danionella* models currently include a third paralog and the lineage-restricted paralogs from other species were not included in subsequent analyses.

**Fig. 1.**
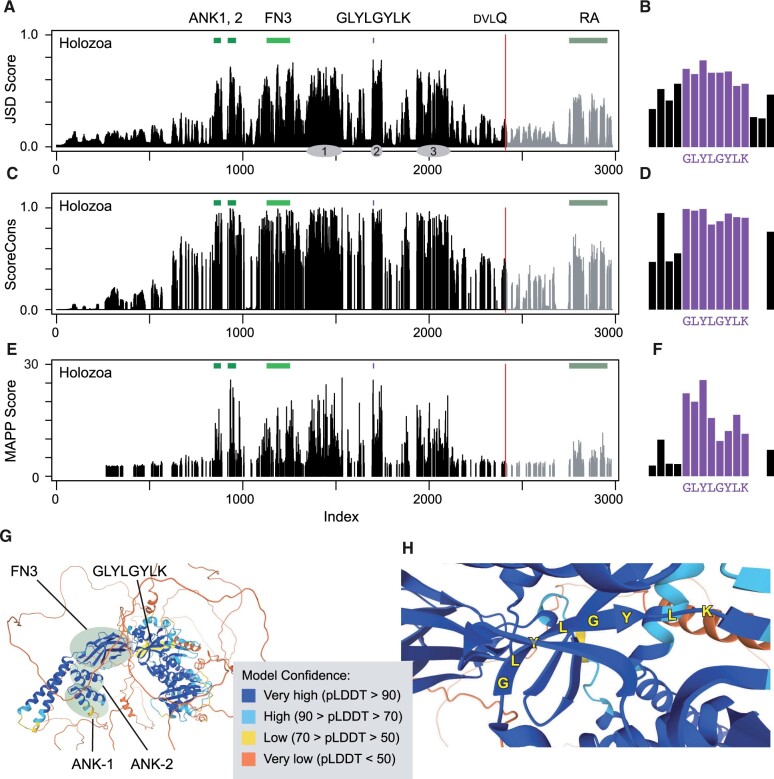
Conserved features of *Ankfn1* homologs. For each histogram a–f) a higher bar indicates higher conservation or constraint at a single residue site for an alignment of Holozoan ANKFN1 proteins. a) Jensen–Shannon divergence (JSD) scores standardized to a range of zero to one. Colored bars above the histogram indicate the positions of the two ANK repeats (ANK1, 2) and FN3 domains, the conserved GLYLGYLK peptide, and the RA domain of ancestral paralogs. A vertebrate-conserved DVLQ peptide (red line) marks the carboxyterminal extent of homology between ancestral and derived paralogs. Gray ovals indicate approximate positions of the three conserved nonmotif regions of Zhang et al. (2015). b) JSD scores at the conserved peptide, *y*-axis same as (a). c) ScoreCons residue conservation using Valdar’s scoring method. d) ScoreCons scores at the conserved peptide, *y*-axis same as (c). e) MAPP median score for substitution at each site. f) MAPP scores at the conserved peptide, *y*-axis same as (e). g) AlphaFold2-predicted structure for mouse ANKFN1 protein, showing positions of the ANK, FN, and GLYLGYLK (yellow highlight) regions and confidence scores. h) Detailed view of the structure in (g) showing the predicted GLYLGY beta strand in relation to flanking strands.

We analyzed ANKFN1 protein sequence conservation with several tools. We first curated 208 homologous protein sequences from 159 diverse Holozoan organisms to represent full or near-full gene models across the well-conserved gene genealogy and performed alignments in MUSCLE (Edgar 2004a, 2004b). Protein sequence models that performed poorly in multiple sequence alignment relative to sister groups and potential distant homologs in a Euglenoid and two Mucomycote fungi were excluded. Jensen–Shannon divergence scores (Capra and Singh 2007) highlighted both known motifs and nonmotif-conserved blocks (Fig. 1a). The most-conserved site in this analysis was the first tyrosine residue in the GLYLGYLK peptide (Fig. 1b) and six of the eight residues in the peptide were among the most-conserved 2% of sites in the alignment. Similarly, a sequence-weighted composite score (Valdar 2002) (Fig. 1c) placed three of the eight GLYLGYLK residues in the most-conserved 2% (Fig. 1d). MAPP (Stone and Sidow 2005) was used to characterize physicochemical constraints based on the amino acid representation and variability. Plotting the median substitution score (Fig. 1e) again highlighted the GLYLGYLK sequence, with the consecutive GLY residues each in the top 2% of scores (Fig. 1f) and two additional residues in the top 5%. This was the only sequence in the Holozoan ANKFN1 alignment with three consecutive positions each in the top 5% of scores.

The predicted structure of human and mouse ANKFN1 proteins by AlphaFold2 (Jumper et al. 2021; Tunyasuvunakool et al. 2021) is further instructive. The conserved GLYLGYLK peptide is predicted to reside in a compact structure of which it is one of the earlier (carboxyterminal) sequence elements (Fig. 1g). The GLYLGY residues are predicted with high confidence to form a beta sheet internal to this structure and to interact with flanking beta sheets as structural components (Fig. 1h). Disruption of this site might be expected to be highly deleterious to ANKFN1 function. Paralog-specific alignments from 49 species for which both were available showed strong conservation of the RA domain among ancestral homologs but lower apparent conservation for C-terminal sequences among derived paralogs (Supplementary Fig. 1). Consistent with both the high degree of conservation and predicted structural requirement, previous mutations at the GLYLGYLK sequence in both mice and flies were reported as presumptive null alleles (Zhang et al. 2015).

### Simultaneous editing of both zebrafish *Ankfn1* homologs

**Fig. 2.**
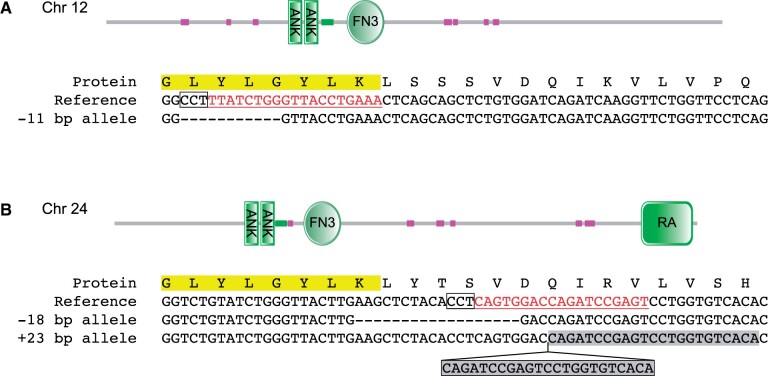
Targeted mutagenesis of *Ankfn1* homologs. Peptide and genomic sequences for targeted sites in *D. rerio Ankfn1* homologs. a) *Ankfn1* on chromosome 12 is a derived paralog that includes two ANK and one FN3 domain but no RA domain. b) *Ankfn1-like* on chromosome 24 is an ancestral paralog including the RA domain. The targeted GLYLGYLK site is highlighted in yellow. CRISPR guide RNA sites are underlined and protospacer adjacent motifs are boxed in each reference sequence. Recovered mutations are shown with deleted base pairs as dashes and insertion of a frame-shifting duplicated sequence (gray shadow) at chromosome 24 shown in a box below the caret.

We targeted the site encoding the GLYLGYLK peptide for the mutagenesis of each paralog because it is highly constrained and resides in a frame-shifting exon, and the homologous site was previously targeted to create phenotypically null alleles in flies and apparent null mutations in mice (Zhang et al. 2015) and should be similarly essential to the function of each zebrafish gene. We coinjected single-guide RNAs targeting the sequence encoding the GLYLGYLK peptide of each *D. rerio* paralog with Cas9 mRNA to allow simultaneous editing at both loci (Fig. 2). Injected G0 founders were outcrossed to wild-type fish and genomic DNA was prepared from G1 offspring to screen for germline-transmitted mutations. Among 60–90 injected offspring, we recovered a single frameshifting 11-bp deletion allele (*sd67*) on chromosome 12 and both an in-frame 18-bp deletion (*sd68*) and a frame-shifting 23-bp insertion allele on chromosome 24 (*sd69*). G1 fish from sequence-verified founders were raised to sexual maturity, genotyped from fin biopsies, and then intercrossed to produce animals for behavioral phenotyping (Talbot and Amacher 2014).

### High penetrance of major phenotypes requires biallelic inactivation of both *Ankfn1* homologs

Initial crosses during allele characterization demonstrated strong effects on vestibular function, swim bladder inflation, and survival to adulthood in animals that had both alleles of both *Ankfn1* paralogs mutated (Tables 1–3). Assessment of gross behavioral and physiological phenotypes in mutant offspring showed a minority of larvae with balance and dorsal orientation deficits, locomotor deficits following tactile stimulation, and subsequent failure of swim bladder inflation. Larvae from these initial observations whose swim bladders failed to inflate died within 2 weeks and were not available for subsequent analysis.

**Table 1.** Cross: Chr12 (−11/+) Chr 24 (+23/+) × Chr12 (−11/+) Chr 24 (−18/+).

Chr12	Chr24	Unaffected	Affected	Total
−11/−11	+23/−18	0	4	4
−11/−11	+23/+	9	0	9
−11/−11	−18/+	5	0	5
−11/−11	+/+	7	1	8
−11/+	+23/−18	7	0	7
−11/+	+23/+	7	0	7
−11/+	−18/+	14	0	14
−11/+	+/+	7	0	7
+/+	+23/−18	4	0	4
+/+	+23/+	8	0	8
+/+	−18/+	4	0	4
+/+	+/+	5	0	5
	Total	77	5	82

**Table 2.** Cross: Chr12 (−11/+) Chr 24 (+23/+) × Chr12 (−11/−11) Chr 24 (+23/+).

Chr12	Chr24	Unaffected	Affected	Total
−11/−11	+23/+23	0	10	10
−11/−11	+23/+	10	0	10
−11/−11	+/+	5	0	5
−11/+	+23/+23	6	0	6
−11/+	+23/+	14	0	14
−11/+	+/+	8	0	8
	Total	43	10	53

**Table 3.** Cross: Chr12 (−11/+) Chr 24 (−18/+) × Chr12 (−11/+) Chr 24 (−18/+23).

Chr12	Chr24	Unaffected	Affected	Total
−11/−11	+23/−18	0	13	13
−11/−11	−18/−18	1	12	13
−11/−11	+23/+	10	0	10
−11/−11	−18/+	9	0	9
−11/+	+23/−18	15	0	15
−11/+	−18/−18	15	0	15
−11/+	+23/+	16	0	16
−11/+	−18/+	25	0	25
+/+	+23/−18	14	0	14
+/+	−18/−18	8	0	8
+/+	+23/+	8	0	8
+/+	−18/+	8	0	8
	Total	129	25	154

**Fig. 3.**
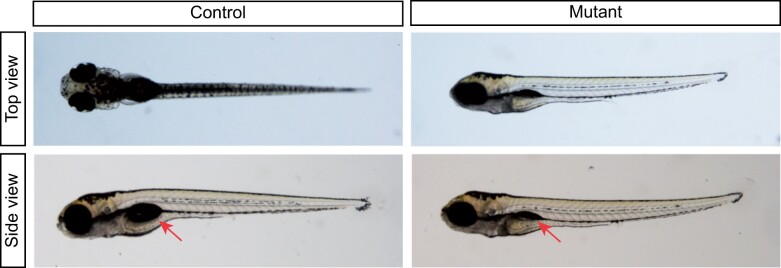
Swim bladder and posture defects in *Chr12/Chr24* mutant zebrafish. By 5 days postfertilization mutant animals frequently failed to maintain dorsal orientation while swimming, with unaffected control animals maintaining a dorsal-up posture while double-mutant animals frequently held a rotated posture, with dorsoventral axis at a skew angle or fully to the side (top view). Swim bladders typically failed to inflate in mutant animals (side view, red arrows).

We then set up formal dihybrid crosses of the frameshifting Chr12 and Chr24 mutant alleles in two cohorts (Table 4). Fish with both genes inactivated displayed abnormal balance, abnormal escape response to touch, and subsequent failure of swim bladder inflation (Fig. 3, side view). Double mutant animals failed to maintain a dorsal-up posture (Fig. 3, top view). Progeny were sorted categorically as affected or unaffected by swim bladder inflation, which requires prior vestibular function (Riley and Moorman 2000; Lindsey et al. 2010), and genotyped by PCR.

**Table 4.** Cross: Chr12 (−11/+) Chr 24 (+23/+) × Chr12 (−11/+) Chr 24 (+23/+).

Chr12	Chr24	Unaffected	Affected	Total
−11/−11	+23/+23	1	15	16
−11/−11	+23/+	32	5	37
−11/−11	+/+	17	2	19
−11/+	+23/+23	29	0	29
−11/+	+23/+	56	1	57
−11/+	+/+	27	1	28
+/+	+23/+23	12	0	12
+/+	+23/+	35	2	37
+/+	+/+	19	0	19
	Total	228	26	254

Genotype ratios of the offspring did not deviate from Mendelian expectations at 5 days postfertilization (Chi square *P* = 0.73), suggesting good viability until ascertainment. Among 254 offspring of the dihybrid crosses, we observed that all double mutants, with one exception, failed to inflate their swim bladders within 5 days postfertilization (94%). Comparatively, the frequency of inflation failure among all other genotypes was low, 11 out of 238 animals (4.6%). Interestingly, among the symptomatic animals that were not double mutants, the majority (7/11) were homozygous mutant for the Δ11 allele on chromosome 12, the derived copy that lacks the RA domain.

Pooling data from all crosses allowed an assessment of penetrance for each genotype class (Table 5). Categorical phenotypes in double mutant animals showed 96% (54/56) penetrance after combining results from both Chr24 mutations. For Chr12 homozygotes with at least one wild-type allele at Chr24 penetrance was 7% (8/112), including three affected animals that were wild type at Chr24. Only four of the remaining 375 offspring had any observed phenotype and those four did not congregate by genotype class after correcting for sample sizes of the subgroups, with no affected offspring among 110 that had both alleles at Chr24 mutated but at least one wild-type allele at Chr12. This analysis supports a low penetrance of the Chr12 (derived) locus alone and strong synthetic interaction with the Chr24 (ancestral) locus for the behavioral and swim bladder inflation phenotypes.

**Table 5.** Summary of all crosses.

Chr12	Chr24	Unaffected	Affected	Total	Penetrance
−11/−11	+23/+23	1	25	26	0.96
−11/−11	+23/−18	0	17	17	1.00
−11/−11	−18/−18	1	12	13	0.92
−11/−11	+23/+	61	5	66	0.08
−11/−11	−18/+	14	0	14	0.00
−11/−11	+/+	29	3	32	0.09
−11/+	+23/+23	35	0	35	0.00
−11/+	+23/−18	22	0	22	0.00
−11/+	−18/−18	15	0	15	0.00
−11/+	+23/+	93	1	94	0.01
−11/+	−18/+	39	0	39	0.00
−11/+	+/+	42	1	43	0.02
+/+	+23/+23	12	0	12	0.00
+/+	+23/−18	18	0	18	0.00
+/+	−18/−18	8	0	8	0.00
+/+	+23/+	51	2	53	0.04
+/+	−18/+	12	0	12	0.00
+/+	+/+	24	0	24	0.00
	Total	477	66	543	0.12

## Discussion

What happens to paralogous copies after gene duplication across broad time scales is an interesting general question, for which *Ankfn1* is an interesting specific example. All invertebrate lineages examined have one copy, except urochordates, which have none. All vertebrate lineages have both an ancestral *Ankfn1* homolog (including the RA domain) and a derived *Ankfn1* homolog (lacking the RA domain), except therian mammals, which have lost the ancestral copy. The two-homolog arrangement has been conserved across nontherian lineages since it arose between the most recent common ancestor of jawed vertebrates ∼465 MYA and the most recent common ancestor of all vertebrates ∼615 MYA (Kumar et al. 2017). The single ancestral gene is essential in *Drosophila*, while the single derived gene impacts vestibular and neurological phenotypes in mice.

We used *D. rerio* as a model vertebrate for a first look at *Ankfn1* genetic properties in a vertebrate with paralogous copies. We directed mutations to the most highly conserved *Ankfn1* domain of each gene and recovered presumptive null frameshift alleles. By design, these mutations mirrored alleles previously reported for single homologs in mice and flies (Zhang et al. 2015). While evolution acts on a much finer scale than laboratory phenotypes and single locus deletions may have subtler or less-penetrant phenotypes than we had power to see, we found that biallelic inactivation of both Chr12 and Chr24 *Ankfn1* homologs was required to produce striking phenotypes with near complete penetrance. Inactivation of the Chr12 (derived) paralog alone was sufficient for weak penetrance of the larval and swim bladder phenotypes (7% of offspring), while inactivation of the Chr24 (ancestral) paralog had no detected effect on these phenotypes (0/110).

Our analysis supports functional overlap of *Ankfn1* paralogs in normal development of zebrafish vestibular function, consistent with the single derived copy in mice. We found high penetrance for vestibular-related phenotypes in fish larvae homozygous for inactivating mutations in each paralog, but not in other genotype combinations. The present study is limited in that we did not perform detailed phenotyping of inner ear structures or perform expression level or localization measurements to determine whether disruption of one paralog resulted in increased expression of the other as a form of dosage compensation (Diss et al. 2014). Whether more subtle phenotypes occur with each mutant singly will require further investigation. We had limited power to assess the potential for low-penetrance phenotypes in some genotype combinations.

Few examples of paralogous gene pairs in zebrafish for which disruption of both copies creates a phenotype that differs from knockout of either paralog alone have been published (Kettleborough et al. 2013). Our results provide a striking example, with a gross morphological phenotype arising from the disruption of both members of a paralogous gene pair without an overt phenotype penetrant in single disruption of either member alone. By generating single and double mutations, we demonstrated substantial genetic redundancy between paralogous *Ankfn1* genes in zebrafish, despite loss of the RA domain in the derived copy and maintenance of independent paralogs across most lineages during roughly half a billion years of vertebrate evolution.

## Supplementary Material

jkab446_Supplemental_Data_File_S1

jkab446_Supplemental_Data_File_S2

jkab446_Supplemental_Data_File_S3

jkab446_Supplemental_Data_File_S4

jkab446_Supplemental_Data_File_S5

jkab446_Supplemental_Legends

jkab446_Supplemental_Figure_S1

jkab446_Supplemental_Table_S1

jkab446_Supplemental_Table_S2

jkab446_Supplemental_Table_S3

## Data Availability

The authors affirm that all data necessary for confirming the conclusions of the article are present within the article, figures, and tables. Strains are available upon request to NCC. Supplementary Table 1 details the species and sequences included in the ANKFN1 protein conservation analysis. Supplementary Table 2 details the template and primer sequences used to generate sgRNA reagents. Supplementary Table 3 details the primer sequences used for PCR genotyping. Zebrafish *Ankfn1* (chromosome 12) and *Ankfn1-like* (chromosome 24) alleles have been deposited in GenBank with accession numbers OL702935 (*sd67*), OL702936 (*sd68*), and OL702937 (*sd69*). Supplemental material is available at *G3* online.
