# Supplementary material for: Ankfn1-mutant vestibular defects require loss of both ancestral and derived paralogs for penetrance in zebrafish
Source: G3 (Bethesda). 2021 Dec 25;12(3):jkab446. doi: 10.1093/g3journal/jkab446 (PMC9210315; doi:10.1093/g3journal/jkab446)
Supplement: jkab446_Supplemental_Legends [file jkab446_supplemental_legends.docx]

**Supplemental Legends**

**Supplemental Figure S1. Paralog-specific conservation of vertebrate *Ankfn1* homologs.** (**A**) Histogram of JSD scores for an alignment of Holozoan homologs with positions of annotated features repeated from Figure 1A for comparison. (**B**) JSD scores for aligned sites among 98 ANKFN1 protein homologs from 49 distinct species of non-therian jawed vertebrates for which both paralogs passed nominal filters for completeness showed higher scores and more compact alignment than the full Holozoa set due to reduced sequence diversity. Paralogs diverge after the DVLQ peptide (red line), indicated by gray histogram after this point in the joint alignment. Separate alignment of ancestral paralogs showed conservation within the RA domain broadly consistent with other domains. Derived paralogs showed less conservation after the DVLQ point among derived paralogs.

**Supplemental files**

Supplemental Data S1. ANKFN1 protein homologs in FASTA format.

Supplemental Data S2. Holozoa ANKFN1 protein sequence alignment.

Supplemental Data S3. Non-therian vertebrate ANKFN1 protein sequence alignment.

Supplemental Data S4. Non-therian vertebrate ANKFN1 ancestral paralog protein sequence alignment.

Supplemental Data S5. Non-therian vertebrate ANKFN1 derived paralog protein sequence alignment.

Supplemental Figure S1. Paralog-specific conservation of vertebrate *Ankfn1* homologs.

Supplemental Table S1. ANKFN1 protein accessions.

Supplemental Table S2. sgRNA designs.

Supplemental Table S3. Genotype assays.
