# Supplementary figures and images for: Ankfn1-mutant vestibular defects require loss of both ancestral and derived paralogs for penetrance in zebrafish
Source: G3 (Bethesda). 2021 Dec 25;12(3):jkab446. doi: 10.1093/g3journal/jkab446 (PMC9210315; doi:10.1093/g3journal/jkab446)

**A****Holozoa**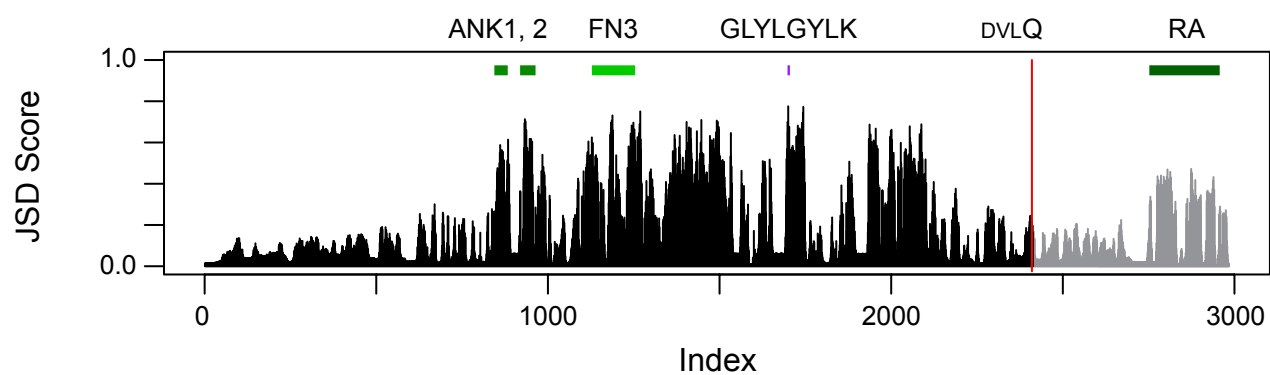**B****Non-Therian Vertebrates**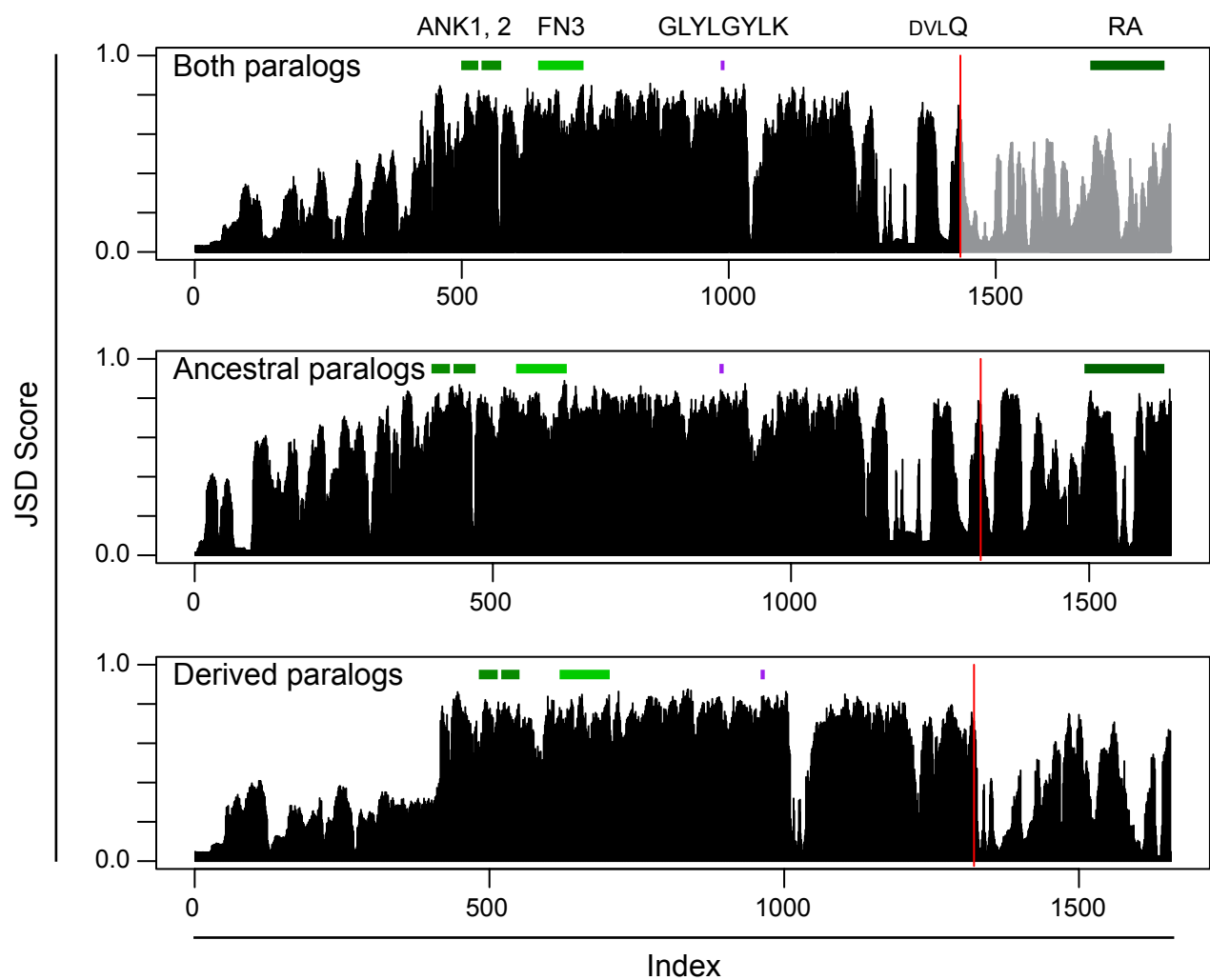

Supplement: jkab446_Supplemental_Figure_S1 [file jkab446_supplemental_figure_s1.pdf]
